# Quantifying Child Mortality Reductions Related to Measles Vaccination

**DOI:** 10.1371/journal.pone.0013842

**Published:** 2010-11-04

**Authors:** Jeremy D. Goldhaber-Fiebert, Marc Lipsitch, Ajay Mahal, Alan M. Zaslavsky, Joshua A. Salomon

**Affiliations:** 1 Centers for Health Policy and Primary Care and Outcomes Research, Department of Medicine, Stanford University School of Medicine, Stanford, California, United States of America; 2 Program in Health Decision Science, Harvard School of Public Health, Boston, Massachusetts, United States of America; 3 Department of Epidemiology, Harvard School of Public Health, Boston, Massachusetts, United States of America; 4 Department of Immunology and Infectious Disease, Harvard School of Public Health, Boston, Massachusetts, United States of America; 5 Department of Global Health and Population, Harvard School of Public Health, Boston, Massachusetts, United States of America; 6 Department of Health Care Policy, Harvard Medical School, Boston, Massachusetts, United States of America; University of Oxford, Viet Nam

## Abstract

**Background:**

This study characterizes the historical relationship between coverage of measles containing vaccines (MCV) and mortality in children under 5 years, with a view toward ongoing global efforts to reduce child mortality.

**Methodology/Principal Findings:**

Using country-level, longitudinal panel data, from 44 countries over the period 1960–2005, we analyzed the relationship between MCV coverage and measles mortality with (1) logistic regressions for no measles deaths in a country-year, and (2) linear regressions for the logarithm of the measles death rate. All regressions allowed a flexible, non-linear relationship between coverage and mortality. Covariates included birth rate, death rates from other causes, percent living in urban areas, population density, per-capita GDP, use of the two-dose MCV, year, and mortality coding system. Regressions used lagged covariates, country fixed effects, and robust standard errors clustered by country. The likelihood of no measles deaths increased nonlinearly with higher MCV coverage (ORs: 13.8 [1.6–122.7] for 80–89% to 40.7 [3.2–517.6] for ≥95%), compared to pre-vaccination risk levels. Measles death rates declined nonlinearly with higher MCV coverage, with benefits accruing more slowly above 90% coverage. Compared to no coverage, predicted average reductions in death rates were −79% at 70% coverage, −93% at 90%, and −95% at 95%.

**Conclusions/Significance:**

40 years of experience with MCV vaccination suggests that extremely high levels of vaccination coverage are needed to produce sharp reductions in measles deaths. Achieving sustainable benefits likely requires a combination of extended vaccine programs and supplementary vaccine efforts.

## Introduction

Historically, measles infections have been a major cause of morbidity and mortality in children, even though effective measles containing vaccines (MCV) were first developed more than 40 years ago [1]. Global efforts to prevent measles deaths have aligned around the United Nations' Millennium Development Goals (MDG), which identify childhood mortality reduction as a key priority, specifically calling for a two-thirds reduction in mortality among children under age 5, between 1990 and 2015 (MDG 4). MCV vaccination programs form a cornerstone of these efforts with ambitious targets, including universal childhood measles vaccination and 90% reductions in measles deaths from year 2000 levels (733,000 worldwide) [2].

Thus far, substantial progress has been made. In 2008, there were an estimated 164,000 measles deaths, a 78% reduction compared to mortality in 2000 [2]. However, the continued effectiveness of vaccination efforts depends on the interaction of numerous socio-demographic and systemic factors that define patterns of measles transmission, effectiveness of vaccination programs, and survival of infected children. In light of these challenges, it is important to monitor program scale-up and gauge whether advances in vaccination coverage will be sufficient to attain global targets for reducing measles mortality [3], [4], [5], [6].

As efforts proceed to expand coverage and sustain the benefits of vaccination in countries with the highest burdens of measles mortality, our aim in this study was to quantify the relationship between historical MCV coverage increases and reductions in child mortality in light of related demographic and economic factors, and critically consider the implications of our findings for current vaccination efforts in high-burden settings.

## Methods

We used regression analyses to assess the relationship between MCV coverage and measles mortality in children under five years, conditioning on relevant country-level factors. The regression models related changes in measles mortality in children under 5 years of age to changes in MCV coverage levels over time within each country while adjusting for country-level variables. Vaccination coverage is expected to influence measles incidence, which in turn will impact on measles mortality, through the mediating effect of the case fatality rate (CFR). Because epidemiological targets are expressed in terms of mortality reductions, and because accurate measles incidence data are difficult to obtain for many countries, we chose to model a reduced-form relationship between MCV coverage and mortality. By examining the coverage-mortality relationship within countries and over time and by including country-level variables that are likely to influence incidence, CFR, or both, our aim was to minimize potential confounding in characterizing the relationship between MCV and mortality.

### Model variables

The primary outcome of interest was the measles-specific death rate for children under five years, computed as the number of deaths classified as measles divided by the at-risk population (i.e. children under 5) in a given country and year. The main independent variable was the proportion of 1–2 year-olds vaccinated with at least one dose of MCV during the year. We hypothesized that: (a) higher MCV coverage is strongly related to lower measles mortality, although the relationship will be non-linear, consistent with herd immunity [7]; (b) the likelihood of having no measles deaths in a country-year increases as MCV coverage increases, consistent with stochastic fade-out and local eradication [8].

Other covariates were included in the models because of their direct or indirect relationships with measles mortality or their potential to modify the relationship between MCV coverage and measles mortality. We included an indicator of whether a country-year's vaccination schedule recommended two MCV doses. Two-dose vaccination was introduced to increase immunity to measles infection [9], [10]. Therefore, a given level of MCV coverage with a two-dose course could lead to greater reductions in measles mortality than with a single dose.

We included changes in determinants of infectious disease epidemic dynamics: population density, proportion living in urban areas, and crude birth rate in a given country and year. Higher population density increases contact rates and transmission. Greater urbanization increases contact rates for urban dwellers, but also reduces travel distances to medical facilities. Its net effect on measles mortality is unclear. Higher birth rates increase the influx of susceptible individuals leading to greater epidemic risk.

The models included changes in socioeconomic determinants of health over time: real per-capita gross domestic product (GDP) (2000 international dollars) and mortality rates from all causes other than measles in children under five years. Increasing GDP is associated with reduced sickness and improved survival, likely related to improvements in education, nutrition, public health, and health care [11], [12], [13], [14].

Year and mortality coding system (International Classification of Disease (ICD) version) were included to enhance outcome comparability over time. Observation year reflects secular trends not captured by other variables. Changes in ICD coding system can cause otherwise similar deaths to be classified differently, artificially changing apparent measles mortality.

### Data sources and inclusion criteria

The WHO Mortality Database provided age-specific death rates by cause, and with a specified coding system, collected in a common format [15]. A previous study [16] provided quality ratings for each country's mortality data. Vaccine coverage after 1980 was derived from WHO/UNICEF estimates [17]. WHO/UNICEF estimates were used instead of Demographic and Health Surveys (DHS) since, although DHS vaccination data are often used in analyses of vaccination [18], [19], they are not available for most countries that we were able to evaluate in this analysis. For vaccine coverage prior to 1980, we relied on country-specific reports (Section S1 in Appendix S1). WHO provided data on the year in which a country introduced a two-dose MCV schedule [20]. Other data were derived from the World Bank [21], United Nations Population Division [22], and the Penn World Tables [23]. For 1960–2005, countries were only included in the analysis if they had country-years with medium or high-quality mortality data, and MCV coverage as well as all other covariates were available (Section S1 in Appendix S1).

### Statistical methods

Data analysis was undertaken using longitudinal panel regressions. Logistic regression was used to assess the odds of having no measles deaths in under-5 children in a given country-year compared to pre-vaccination risk levels. Linear regression was used to model the log death rate from measles in a given country-year (Section S2 in Appendix S1). In all models, aside from observation year, all independent variables that are not indicators or percentages were log-transformed so estimated coefficients could be interpreted as elasticities [24]. For example, the model coefficient for MCV coverage may be understood as the percent change in measles mortality rates for children under 5 associated with a 1% change in coverage. Independent variables other than observation year and ICD coding system were lagged by 1 year. Country-level fixed effects were included to absorb unobserved country-level heterogeneity (e.g., determinants of variation in case fatality rates not explained by other covariates in the model), and robust standard errors, clustered by country, were estimated using the jackknife method.

Since the relationship between coverage and mortality is not necessarily linear, coverage was either categorized using indicator variables or entered as restricted cubic splines [25]. We divided MCV coverage into six categories: 0%; 1–59%; 60–79%; 80–89%; 90–94%; and ≥95% coverage. These divisions were prospectively defined so the numbers of observations in each level above 0% were nearly equal and cutoffs were divisible by 5. The restricted cubic spline specification used the same cutoffs (Section S2 in Appendix S1).

To translate the spline regression results into interpretable mortality/coverage relationships for different starting MCV coverage levels, we estimated the expected percent reduction in under-5 measles-specific mortality and associated confidence intervals with simulations. The simulations comprised 20,000 random draws of all model coefficients from multivariate normal distributions using the estimated regression coefficients and their associated variance-covariance matrix. With each set of coefficients, we calculated expected measles-specific mortality rates at MCV coverage levels from 1% to 99%. Then, to reflect the change in measles mortality related to increases in coverage, we calculated reduction in measles-specific mortality associated with increasing MCV coverage to 90–99% from coverage levels of 50–95%. We similarly calculated interquartile ranges and 5th and 95th percentiles.

We examined the sensitivity of our results to key assumptions. The restricted cubic spline model specification was compared to: 1) a model without MCV coverage; and 2) a model with a constant log-linear relationship between MCV coverage and mortality. Alternative specifications were compared using Akaike and Bayesian Information Criteria [26], [27]. We also used these Information Criteria to assess alternative categorizations of MCV coverage levels. We assessed the impact of lagging MCV coverage by one year compared to a model in which MCV coverage was averaged over the previous 5 years. We assessed the impact of non-linear time trends by comparing the main model with one using year fixed effects. We explored other statistical models including negative binomial regressions. To consider the potential relevance of the historical patterns to countries with large measles burden at present, we assessed potential bias by reanalyzing subsets of countries from our full dataset with lower and higher per-capita GDP.

All analyses were undertaken using Stata/SE 10.0 (StataCorp, College Station TX).

## Results

The countries and years included in our analysis spanned broad ranges in terms of measles mortality, birth rate, urban population and density, and MCV coverage (Table 1). Countries were mostly middle- to high-income, as the analysis required higher-quality vital registration systems. Nonetheless, all MCV coverage levels were represented, with MCV coverage above 60% in the majority of years.

**Table 1:** Characteristics of the study sample.*

	Values
	(n = 980)
ICD-7 coding system used, % of country-years	18.4
ICD-8 coding system used, % of country-years	19.7
ICD-9 coding system used, % of country-years	55.4
ICD-10 coding system used, % of country-years	6.5
High quality mortality data, % of country-years)	31.7
Measles death rate, per 100,000 children under 5 per year, mean (SD)	7.0 (29.3)
No measles deaths observed, % of country-years	38.7
Background mortality rate, per 100,000 children per year, mean (SD)	1,291.1 (1,289.3)
MCV coverage, % of 12–24 month-olds, mean (SD)	57.4 (39.6)
MCV coverage of 1–59%, % of country-years	12.6
MCV coverage of 60–79%, % of country-years	14.7
MCV coverage of 80–89%, % of country-years	14.2
MCV coverage of 90–94%, % of country-years	14.7
MCV coverage of ≥95%, % of country-years	17.3
Two doses of MCV, % of country-years	30.4
Crude birth rate, per 1,000 adults per year, mean (SD)	19.0 (9.1)
Population density, per sq km, mean (SD)	102.3 (100.5)
Under-5 population, millions, mean (SD)	2.8 (4.5)
Urban, % of population, mean (SD)	67.4 (14.5)
Per-capita GDP, in 2000 international dollars, mean (SD)	12,853 (7,629)

*Countries included in the analysis: Austria, Azerbaijan, Belarus, Belgium, Belize, Brazil, Bulgaria, Canada, Chile, Colombia, Costa Rica, Cuba, Denmark, El Salvador, Finland, France, Germany, Guatemala, Hungary, Ireland, Israel, Italy, Kazakhstan, Kuwait, Kyrgyzstan, Luxembourg, Mexico, Netherlands, Norway, Panama, Republic of Korea, Romania, Russian Federation, Spain, Sweden, Switzerland, TFYR Macedonia, Turkmenistan, Ukraine, United Kingdom, United States of America, Uruguay, Uzbekistan, and Venezuela.

Higher MCV coverage was associated with a greater chance of having no measles deaths in children under 5 in a given year (Table 2). Coverage levels ≥80% were significantly and positively associated with no measles deaths in the multivariable model. Without MCV vaccination, the model predicted that 5% of country-years would be free of measles deaths for children under 5 years, increasing to 69% of country-years when MCV coverage exceeded 95%.

**Table 2:** Results from logistic regression model for having no measles deaths in children under 5 years.*

Independent variables	Odds ratio	95% confidence interval	P-value
MCV coverage of 1–59%	2.743	[0.32–23.27]	0.345
MCV coverage of 60–79%	6.009	[0.66–54.38]	0.108
**MCV coverage of 80–89%**	**13.831**	**[1.56–122.67]**	**0.020**
**MCV coverage of 90–94%**	**18.863**	**[2.05–173.32]**	**0.011**
**MCV coverage of ≥95%**	**40.665**	**[3.19–517.59]**	**0.005**
ICD-8 coding system	0.390	[0.05–3.13]	0.365
ICD-9 coding system	0.330	[0.02–6.76]	0.461
ICD-10 coding system	0.108	[0.00–3.90]	0.216
Year	1.099	[0.96–1.26]	0.164
Two doses of MCV	1.272	[0.52–3.10]	0.588
Crude birth rate	0.045	[0.00–13.31]	0.276
Urban	0.911	[0.74–1.13]	0.381
Population density	13.768	[0.00–439,572.00]	0.611
Per-capita GDP	5.361	[0.18–160.86]	0.324
Background mortality rate	0.670	[0.32–1.40]	0.279

*Observations: 878; Countries: 38; Observations per country (min: 5; avg: 23.1; max: 43); F: 9.2; p: <0.0001.

6 countries accounting for 102 observations could not be included in the logistic regression because all years had no measles deaths (Belarus and Luxembourg) or all years had measles deaths (Guatemala, Romania, Turkmenistan, and Venezuela).

All variables are use indicators except year which is expressed as calendar year, urban which is expressed percentage, and crude birth rate, population density, per-capita GDP, and background mortality rate which are all log-transformed continuous variables.

The comparator for the odds ratios are country-years with 0% MCV coverage using ICD-7 coding systems without a two-dose MCV course.

Coverage levels above 60% were associated with increasingly significant reductions in measles mortality in the multivariable model. Table 3 shows the relationship between MCV coverage and measles death rates in children under 5. Measles mortality declined most rapidly as MCV coverage increased from 30% through 75% (from −26% to −83% over this range), while at MCV coverage above 85%, further mortality declines were significant but smaller in scale (an additional −5% from 85% to 99% coverage).

**Table 3:** Results from linear regression model for logged measles-specific death rates for children under 5 years.*

Independent variables	Coefficient	95% confidence interval	P-value
MCV coverage of 1–59%	−0.236	[−1.06–0.59]	0.568
**MCV coverage of 60–79%**	**−1.639**	**[−2.95– −0.32]**	**0.016**
**MCV coverage of 80–89%**	**−2.298**	**[−3.55– −1.05]**	**0.001**
**MCV coverage of 90–94%**	**−2.576**	**[−3.85– -1.30]**	**0.000**
**MCV coverage of ≥95%**	**−2.924**	**[−4.18– −1.67]**	**0.000**
ICD-8 coding system	0.499	[−0.33–1.33]	0.232
ICD-9 coding system	0.731	[−0.95–2.41]	0.386
ICD-10 coding system	1.321	[−0.63–3.28]	0.180
**Year**	**−0.117**	**[−0.22– −0.01]**	**0.028**
Two doses of MCV	−0.396	[−1.28–0.48]	0.370
**Crude birth rate**	**3.450**	**[0.57–6.33]**	**0.020**
Urban	0.078	[−0.02–0.17]	0.108
Population density	−1.302	[−4.03–1.42]	0.340
Per-capita GDP	−0.464	[−2.29–1.37]	0.611
Background mortality rate	0.300	[−1.68–2.28]	0.762
Constant	223.175	[17.16–429.19]	0.034

*Observations: 980; Countries: 44; Observations per country (min: 5; avg: 22.3; max: 43); R-squared (within: 0.62; between: 0.33; overall: 0.46); F: 129.84; p: <0.0001.

All variables are use indicators except year which is expressed as calendar year, urban which is expressed percentage, and crude birth rate, population density, per-capita GDP, and background mortality rate which are all log-transformed continuous variables.

The comparator for the odds ratios are country-years with 0% MCV coverage using ICD-7 coding systems without a two-dose MCV course.

Both observation year and crude birth rate also were significantly associated with measles death rates (Table 3). There was an expected 12% decline in measles deaths each year, holding all other model variables fixed. Higher birth rates were associated with higher measles mortality rates, with a 1% increase in birth rate associated with a 3.5% increase in measles death rate. This is consistent with the observation that faster entry of susceptible individuals into the population makes measles outbreaks more likely.

Notably, though not reaching statistical significance at the p<0.05 level, results were suggestive that the introduction of a 2-dose measles vaccination course were related to a greater chance of having no measles deaths in a given year and to lower measles mortality levels in children under 5 years of age (Table 2 and Table 3).

**Figure 1:**
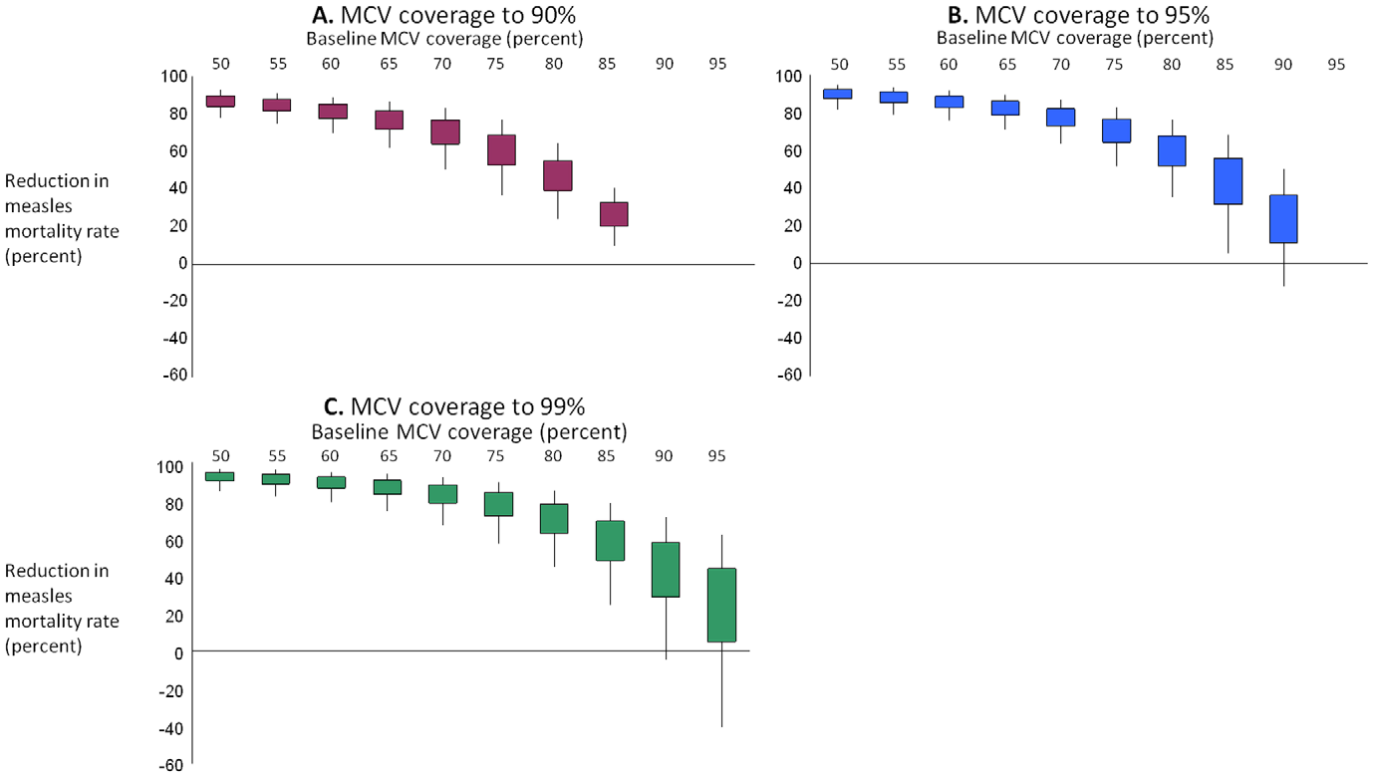
Estimated changes in measles-specific mortality rates for children under 5 years old with increasing MCV coverage. Shown in the graph is the percentage change in measles-specific mortality rates in children under 5 years associated with increasing MCV coverage to 90% (Panel A), 95% (Panel B), and 99% (Panel C) from different starting MCV coverage levels (solid colored bars – interquartile ranges; black vertical lines – 5th to 95th percentile ranges).

While substantial gains are expected with increased MCV coverage, the expected magnitude of additional gains are less certain at coverage levels above 90%. Figure 1 shows the expected impact of increasing MCV coverage from various starting levels to 90% (Panel A), 95% (Panel B), or 99% (Panel C). For example, increasing from 75% to 90% MCV coverage reduces measles mortality rates by 60% (interquartile range: 53–69%; 5th to 95th percentile: 36–77%).

The main findings were assessed in sensitivity analyses (Sections S3, S4, S5, and S6 in Appendix S1). The findings were robust to a range of alternative methodological choices and assumptions, with estimated benefits in the sensitivity analyses differing by less than 15% from those reported in the main analysis. Importantly, because current measles burdens are in countries that are generally poorer than those in our data set, we assessed the associations between MCV and measles mortality for countries stratified by per-capita GDP (above/below $7,000) for countries in our data set. We found that poorer countries had greater benefit from increased MCV coverage, though benefits differed by no more than 5%.

## Discussion

Forty years of international experience with the impact of measles vaccination programs on child mortality suggests that sustained high levels of MCV coverage, along with other factors, contributed to dramatic declines in measles deaths. In light of current efforts to improve child health, measles vaccination has played and will continue to play an important role. As the Measles Initiative reports, impressive reductions have been achieved in some countries with previously high measles mortality rates [28], though there is still much work to be done.

This analysis has several strengths. It characterizes the historical relationship between MCV coverage and mortality as observed across 44 countries over the past 40 years. It evaluates this relationship, conditioning on country differences likely to impact the population dynamics of measles. It captures nonlinearities in the relationship between coverage and mortality, relevant for considering potential health benefits due to increased coverage for countries starting at different MCV coverage levels.

The analysis also has limitations. Data were drawn from multiple sources and rely in part on country –reports on mortality and vaccination coverage. While we cannot rule out bias, the fact that the estimated effects of covariates such as crude birth rate were consistent with their expected directions offers some reassurance. Additionally, we estimated coverage-mortality relationships for countries with higher quality vital registration, adjusted for changes in ICD coding and year, and included country fixed effects. Further, we identified effects of vaccination from distinct patterns of within-country longitudinal variation in vaccination rates, adjusting for a wide array of variables that likely capture important variation across countries. We also found that our results were robust to various methodological choices in a broad array of sensitivity analyses.

The analysis used national estimates of measles deaths and measles coverage, and therefore cannot comment on the effects of sub-national heterogeneity. These effects likely blunt the impact of vaccine coverage on measles reductions, particularly in larger countries. Our use of country fixed-effects and the explicit inclusion of other potential determinants of unequal MCV coverage within countries aimed to mitigate this to a certain extent.

Because of limitations in data availability, our analysis could not include countries from Africa or the Indian subcontinent. Differences between these countries and those in our dataset (for example, high rates of under-nutrition in the former) limit our ability to make precise out-of-sample projections. Additionally, case fatality rates (CFR) may also be higher in lower-income countries not included in our data set [29]. We used country fixed effects and controlled for observation year to account for difference that remained constant across countries or that changed similarly across countries over time. We also conducted sensitivity analyses to assess differences in findings by per-capita GDP level or region. None of these analyses showed evidence of strong bias (Section S1 in Appendix S1), but we suggest caution in extrapolating to specific countries outside of our dataset.

Notwithstanding these limitations, the historical patterns examined in this study do offer important insights relevant to present circumstances. Specifically, they suggest that very high coverage levels, perhaps well above 95%, sustained over substantial periods of time, are likely needed to achieve the types of reductions observed historically. Furthermore, our main results suggest that the use of second doses of MCV — as an example of a vaccination strategy that can complement higher coverage levels — could be important, especially in countries where higher levels of malnutrition, greater incidence of diarrhea, and resulting weaker immunologic responses to vaccines among children are more common.

While our goals in this study were to use historical data to estimate a relationship between MCV coverage and measles mortality, comparison to theoretical models is also appropriate. Without vaccination, years with large-scale epidemics alternate periodically with years of relatively low measles incidence [30], [31]. As vaccination increases, long-run average measles incidence declines [31]. Through herd immunity – indirect protection of unvaccinated individuals due to interrupted chains of transmission – measles may theoretically be eradicated below 100% vaccination – implying percent reductions greater than MCV coverage levels. As the proportion of the population vaccinated may be larger than the proportion achieving immunity because of issues like spoilage, the opposite may also be true [32]. Measles mortality – a stated target of the MDGs – is related to vaccination via incidence multiplied by the case fatality rate. With a constant CFR, mortality should respond to MCV coverage increases like incidence. However, CFR depends on a number of factors [29]. For example, vaccination increases the average age of infection [31]. If older children are more likely to survive, then mortality may decline more than incidence.

Results from this analysis are broadly consistent with theoretical relationships and previous studies. While we do not directly observe information on interruption in chains of infection transmission at different coverage levels, we interpret the non-linearities in the relationship between coverage and mortality as an indication of herd immunity. A study of measles immunity in Europe in the pre-vaccine era found that the theoretical proportion needed to vaccinate at birth to achieve eradication was between 86% and 97%, suggesting modest herd immunity effects [33], [34]. Similarly, we found that the proportion of country-years with no observed measles deaths increased sharply at such coverage levels. Consistent with these findings, we estimated that the benefits of herd immunity are modest with reduction in measles deaths a few percentage higher than MCV coverage levels in the 45–90% coverage range. For MCV coverage below 45%, reductions in deaths actually lag behind increases in coverage. As this finding is consistent with the potential failure of vaccination to provoke sufficient immunity in all children, further emphasis on vaccination program quality, 2-dose MCV courses, and supplemental immunization activities is warranted in countries scaling-up from low coverage levels.

Sustaining high measles coverage has value in developed and developing countries. The majority of measles deaths occurs in resource-poor countries [28]. Simulation studies illustrate the importance of measles vaccination in these populations [6], [35], [36], with estimated benefits similar to our findings. Studies also highlight the importance of program quality and supplemental immunization activities. In developed countries, vulnerability to renewed measles epidemics exists despite longstanding vaccination programs [37].

Measles vaccination is a cornerstone of preventing childhood mortality. In over 40 years of worldwide experience with measles vaccination, impressive reductions in measles incidence and mortality have been achieved through sustained population coverage levels above 90%. Given the challenges of sustaining high measles coverage in countries currently facing large measles burdens, a combination of extended vaccine programs and supplementary vaccine efforts should be pursued.

## Supporting Information

Appendix S1 Supplemental appendix with supporting information.
(0.35 MB PDF)
