# Supplementary material for: Quantifying Child Mortality Reductions Related to Measles Vaccination
Source: PLoS One. 2010 Nov 4;5(11):e13842. doi: 10.1371/journal.pone.0013842 (PMC2973966; doi:10.1371/journal.pone.0013842)
Supplement: Appendix S1 — Supplemental appendix with supporting information. (0.35 MB PDF) [file pone.0013842.s001.pdf]

Quantifying child mortality reductions related to measles vaccination

Jeremy D Goldhaber-Fiebert, Marc Lipsitch, Ajay Mahal, Alan M Zaslavsky, Joshua A Salomon

Supporting Information

S1. Data description and coding 2
S2. Variable construction 8
S3. Sensitivity analysis: model fit and specifications 9
S4. Sensitivity analysis: alternative regressions among country subsets defined by income or region 13
S5. Sensitivity analysis: year linear trends versus year fixed effects 15
S6. Country-years with no observed measles deaths 16

Supporting Information S1. Data description and coding

Data used in the analyses were derived from multiple sources (**Table S1A**). Measles deaths were identified from the WHO Mortality Database based on ICD codes shown in **Table S1B**. Measles vaccine coverage estimates for years prior to 1980 were derived from a variety of country-specific reports shown in **Table S1C**. The countries, numbers of observations, and observation years with data present for all variables are shown in **Table S1D**.

Table S1A. Data sources and years for analyses

Variables	Years	Source/Description
Cause/age-specific mortality counts<br>Size of age-specific population at risk<br>Age-specific all other cause death rate	1960-2005	WHO Mortality Database (For measles cause-specific ICD codes used see **Table S1B**)
Quality rating for each country's vital registration data	n/a	Mathers CD, Fat DM, Inoue M, Rao C, Lopez AD (2005) Counting the dead and what they died from: an assessment of the global status of cause of death data. Bull World Health Organ 83: 171-177.
Vaccination coverage	1980-2005	WHO/UNICEF
Vaccination coverage and/or start date of vaccination	1960-1980	Various Source on Vaccination (Complete list of sources in **Table S1C**)
Second dose of MCV used in vaccination	1960-2005	WHO
Crude birth rate<br>Percent urban<br>Population size and structure<br>Population density	1950-2005	United Nations World Population Prospects
Real per-capita GDP (Laspeyres, Constant I$2000)	1950-2004	Penn World Tables 6.2

Abbreviations: ICD, International Classification of Diseases; MCV, measles-containing vaccine; WHO, World Health Organization; GDP, gross domestic product

Table S1B. International Classification of Disease (ICD) codes used to identify measles deaths*

ICD Edition	Detailed List Numbers	Description
ICD-7	085	Measles
ICD-8	055	Measles
ICD-9	055	Measles
ICD-10	B05	Measles
ICD-10	B050	Measles complicated by encephalitis
ICD-10	B051	Measles complicated by meningitis
ICD-10	B052	Measles complicated by pneumonia
ICD-10	B053	Measles complicated by otitis media
ICD-10	B054	Measles with intestinal complications
ICD-10	B058	Measles with other complications
ICD-10	B059	Measles without complication

* Summary codes corresponding to these detailed list numbers were used to extract country, year, and gender-specific mortality counts for measles-related deaths.

Table S1C. Sources for Measles Vaccine Coverage prior to 1980

Sources
• The Belgian Childhood Vaccine Schedule (EUVAC NET). EUVAC NET; [cited 2007 November 13]; Available from: http://www.euvac.net/graphics/euvac/vaccination/belgium.html.<br>• The Bulgarian Childhood Vaccine Schedule (EUVAC NET). EUVAC NET; [cited 2007 November 13]; Available from: http://www.euvac.net/graphics/euvac/vaccination/bulgaria.html.<br>• Canadian National Report on Immunization, 1996 (Public Health Agency of Canada). Public Health Agency of Canada; [cited 2007 November 13]; Available from: http://www.phac-aspc.gc.ca/publicat/ccdr-rmtc/97vol23/23s4/index.html.<br>• Using surveillance data and outbreak investigations to strengthen measles immunization programs (WHO). WHO; [cited 2007 November 13]; Available from: http://www.who.int/vaccines-documents/DocsPDF/www9645.pdf.<br>• The Croatian Childhood Vaccine Schedule. (EUVAC NET). EUVAC NET; [cited 2007 November 13]; Available from: http://www.euvac.net/graphics/euvac/vaccination/croatia.html.<br>• The Czech Childhood Vaccine Schedule (EUVAC NET). EUVAC NET; [cited 2007 November 13]; Available from: http://www.euvac.net/graphics/euvac/vaccination/czechrepublic.html.<br>• Measles, Mumps, Rubella (MMR) Vaccine Discussion Pack (Health Boards Executive). Health Boards Executive; [cited 2007 November 13]; Available from: http://91.186.163.172/hpsc/A-Z/VaccinePreventable/MMR/Publications/File,1234,en.pdf.<br>• National Immunization Data, Republic of Korea (WHO). WHO; [cited 2007 November 13]; Available from: http://www.wpro.who.int/NR/rdonlyres/5E3697A9-FA42-45D5-8301-3B941D8D8CA0/0/Poster_KOR.pdf.<br>• Measles in New Zealand (New Zealand Ministry of Health). New Zealand Ministry of Health; [cited 207 November 13]; Available from: http://www.cdhb.govt.nz/measles/Measles_in_New_Zealand.htm.<br>• The Romanian Childhood Vaccine Schedule (EUVAC NET). EUVAC NET; [cited 2007 November 13]; Available from: http://www.euvac.net/graphics/euvac/vaccination/romania.html.<br>• The Slovak Childhood Vaccine Schedule (EUVAC NET). EUVAC NET; [cited 2007 November 13]; Available from: http://www.euvac.net/graphics/euvac/vaccination/slovakia.html.<br>• The Slovenian Childhood Vaccine Schedule (EUVAC NET). EUVAC NET; [cited 2007 November 13]; Available from: http://www.euvac.net/graphics/euvac/vaccination/slovenia.html.<br>• Measles elimination plan: Spain (Instituto de Salud Carlos III). Instituto de Salud Carlos III; [cited 2007 November 13]; Available from: http://www.euvac.net/graphics/euvac/pdf/plan_spain.pdf.<br>• The Swiss Childhood Vaccine Schedule (EUVAC NET). EUVAC NET; [cited 2007 November 13]; Available from: http://www.euvac.net/graphics/euvac/vaccination/switzerland.html.<br>• Expanded Program on Immunization in Brazil: vaccination coverage. EPI Newsletter. 1979 December;1(4).<br>• Status of immunization programs in the American region, 1978. EPI Newsletter. 1980;2(1).<br>• Status of immunization programs in the American region, 1979. EPI Newsletter. 1980;2(6).<br>• Epidemic outbreak of measles in three central provinces of Chile. EPI Newsletter. 1981;3(2).<br>• Panama: summary of immunization data from 1979 Family Planning/Maternal Child Health Survey. EPI Newsletter. 1981;3(3).<br>• Vaccination coverage and reported cases of diptheria, whooping cough, tetanus, measles, and poliuomyelitis, per 100,000 population (provisional data): region of the Americas. EPI Newsletter. 1981;3(5).<br>• Measles outbreak -- Romania, 1997. MMWR Morb Mortal Wkly Rep. 1997 Dec 12;46(49):1159-63.

- Progress towards measles elimination, WHO Eastern Mediterranean Region, 1980-1998. Wkly Epidemiol Rec. 1999 Dec 17;74(50):434-9.
- Measles outbreak in Kaunas, Lithuania. Euro Surveill. 2002 October;10(44).
- Agocs MM, Markowitz LE, Straub I, Domok I. The 1988-1989 measles epidemic in Hungary: assessment of vaccine failure. Int J Epidemiol. 1992 Oct;21(5):1007-13.
- Burgasov PN, Andzaparidze OG, Popov VF. The status of measles after five years of mass vaccination in the USSR. Bull World Health Organ. 1973;49(6):571-6.
- Cilla G, Basterretxea M, Artieda J, Vicente D, Perez-Trallero E. Interruption of measles transmission in Gipuzkoa (Basque Country), Spain. Euro Surveill. 2004 May;9(5):29-31.
- Galindo M. Cuba's National Immunization Program. [cited 2007 November 13]; Available from: http://www.medicc.org/publications/medicc_review/1004/pages/spotlight.html.
- Goh D, Chew F, Khor S, Lee B. Resurgence of measles in Singapore: profile of hospital cases. J Paediatr Child Health. 1999 Oct;35(5):493-6.
- Gomi H, Takahashi H. Why is measles still endemic in Japan? Lancet. 2004 Jul 24-30;364(9431):328-9.
- Loevoll O, Sandbu S. Measles and measles immunisation in Norway: historical review and present situation. [cited 2007 November 13]; Available from: http://www.eurosurveillance.org/ew/2002/020321.asp#2.
- McFarland JW, Mansoor OD, Yang B. Accelerated measles control in the Western Pacific region. J Infect Dis. 2003 May 15;187 Suppl 1:S246-51.
- Morice A, Carvajal X, Leon M, Machado V, Badilla X, Reef S, et al. Accelerated rubella control and congenital rubella syndrome prevention strengthen measles eradication: the Costa Rican experience. J Infect Dis. 2003 May 15;187 Suppl 1:S158-63.
- Muscat M, Christiansen AH, Persson K, Plesner AM, Bottiger BE, Glismann S, et al. Measles outbreak in the Oresund region of Denmark and Sweden. Euro Surveill. 2006;11(3):E060330 4.
- Orenstein WA. The role of measles elimination in development of a national immunization program. Pediatr Infect Dis J. 2006 Dec;25(12):1093-101.
- Orenstein WA, Papania MJ, Wharton ME. Measles elimination in the United States. J Infect Dis. 2004 May 1;189 Suppl 1:S1-3.
- Prevots DR, Parise MS, Segatto TC, Siqueira MM, dos Santos ED, Ganter B, et al. Interruption of measles transmission in Brazil, 2000-2001. J Infect Dis. 2003 May 15;187 Suppl 1:S111-20.
- Salleras L, Vidal J, Canela J, Jimenez De Anta MT, Pumarola T, Coll JJ, et al. Seroepidemiology of measles in Catalonia (Spain) 1985-1986. Eur J Epidemiol. 1990 Jun;6(2):207-11.
- Samoilovich EO, Yermalovich MA, Semeiko GV, Svirchevskaya EI, Rimzha MI, Titov LP. Outbreak of measles in Belarus, January-June 2006. Euro Surveill. 2006;11(7):E060727 3.
- Santos JI, Nakamura MA, Godoy MV, Kuri P, Lucas CA, Conyer RT. Measles in Mexico, 1941-2001: interruption of endemic transmission and lessons learned. J Infect Dis. 2004 May 1;189 Suppl 1:S243-50.
- Schmid D, Pichler AM, Wallenko H, Holzmann H, Allerberger F. Mumps outbreak affecting adolescents and young adults in Austria, 2006. Euro Surveill. 2006;11(6):E060615 1.
- Seguliev Z, Duric P, Petrovic V, Stefanovic S, Cosic G, Hrnjakovic IC, et al. Current measles outbreak in Serbia: a preliminary report. Euro Surveill. 2007 Mar;12(3):E070315 2.
- Simpson DM, Ezzati-Rice TM, Zell ER. Forty years and four surveys: how does our measuring measure up? Am J Prev Med. 2001 May;20(4 Suppl):6-14.
- Skutlaberg D, Vainio K, Loevoll O. Laboratory surveillance of measles and rubella in Norway (EpiNorth). EpiNorth; [cited 2007 November 13]; Available from: http://www.epinorth.org/artikler/?id=45370.
- Spika JS, Aidyralieva C, Mukharskaya L, Kostyuchenko NN, Mulders M, Lipskaya G, et al. Measles outbreak in the Ukraine, 2005-2006. Euro Surveill. 2006;11(3):E060309 1.

- Stewart-Freedman B, Kovalsky N. An ongoing outbreak of measles linked to the United Kingdom in an ultra-orthodox Jewish community in Israel. Euro Surveill. 2007 Sep;12(9):E070920 1.
- Swartz TA. Prevention of measles in Israel: implications of a long-term partial immunization program. Public Health Rep. 1984 May-Jun;99(3):272-7.

Table S1D. Observations and years by country

Country	Number of observations	First year	Last year
Austria	37	1960	2004
Azerbaijan	8	1995	2002
Belarus	8	1996	2003
Belgium	31	1960	1997
Belize	9	1982	1996
Brazil	21	1979	1999
Bulgaria	13	1992	2004
Canada	17	1960	2000
Chile	28	1960	1996
Colombia	20	1960	1998
Costa Rica	28	1960	1996
Cuba	22	1979	2000
Denmark	27	1960	1994
El Salvador	14	1960	1997
Finland	34	1960	1995
France	35	1960	2003
Germany	13	1992	2004
Guatemala	24	1960	2003
Hungary	26	1971	1996
Ireland	42	1960	2005
Israel	16	1982	1997
Italy	39	1960	2002
Kazakhstan	5	1994	2004
Kuwait	8	1981	1994
Kyrgyzstan	6	1994	1999
Luxembourg	14	1984	1997
Mexico	36	1960	1997
Netherlands	37	1960	1999
Norway	32	1960	1995
Panama	18	1960	1997
Republic of Korea	16	1987	2002
Romania	33	1961	1998
Russian Federation	11	1994	2004
Spain	41	1960	2004
Sweden	27	1960	1996
Switzerland	34	1960	2004
TFYR Macedonia	9	1995	2003
Turkmenistan	5	1994	1998
Ukraine	11	1994	2004
United Kingdom	43	1960	2004
United States of America	40	1960	2001
Uruguay	16	1960	1996
Uzbekistan	8	1994	2003
Venezuela	18	1960	1994

Supporting Information S2. Variable construction

Logarithmic transformation of measles death rates

For analyses of reductions in measles death rates, the rates were log transformed (natural log). For country-years with zero observed measles deaths, the log-transformed rate is undefined. To prevent these observations from being dropped from the analysis, we therefore replaced zero values with the minimum observed rate divided by 10 and then log-transformed all rates. The minimum non-zero observed rate in the dataset occurred in the United States in 1992 and was $5x10^{-3}$ measles deaths per 100,000 children aged 0 through 5. Sensitivity analyses relating to the treatment of zeros and the analysis of measles specific death rates are presented in **Supporting Information S6**.

Categorizing measles vaccination coverage levels

Measles-containing vaccine (MCV) coverage is a continuous variable running from 0 to 100%. Because the relationship between MCV coverage and measles death rates may be non-linear and because we did not wish to impose a functional form, we categorized MCV coverage into a number of discrete levels. We defined these divisions prospectively so that the number of observations in each level above 0% MCV coverage was nearly equal and cutoffs were divisible by 5. MCV coverage was categorized into the following levels: 0%; 1-59%; 60-79%; 80-89%; 90-94%; and >=95% coverage. We also constructed restricted cubic splines for MCV coverage with knots placed at the same cutoffs [See: Luke Keele. *Semiparametric Regression for Social Sciences*. Chichester, England: John Wiley and Sons, Ltd. 2008].

Supporting Information S3. Sensitivity analysis: model fit and specifications

We compared alternative model specifications using both the Akaike Information Criterion (AIC) and the Bayesian Information Criterion (BIC). In general a reduction in the AIC or BIC from one model specification to another of approximately 2 or greater indicates a significant improvement in model fit even after penalization for specifications that include additional parameters.

We compared the following model specifications for MCV coverage: 1) the base case model with all covariates but omitting any MCV coverage term (i.e., *no MCV coverage term*); 2) the base case model with all covariates and MCV coverage a continuous variable ranging from 0-1 with one regression coefficient estimated for this term (i.e., *linear MCV coverage term*); 3) the base case model with all covariates and MCV coverage categorized into separate indicators for each coverage range as described in **Supporting Information S2** (i.e., *indicators MCV coverage terms*); 4) the base case model with all covariates and MCV coverage entered as a series of restricted cubic splines as described in **Supporting Information S2** (i.e., *restricted cubic splines for MCV coverage*). The model specification where MCV coverage enters as restricted cubic splines is preferred as it minimizes both the Akaike Information Criterion (AIC) and the Bayesian Information Criterion (BIC) (**Table S3A**).

Table S3A. Specification of MCV coverage in the model

Specification	AIC	BIC
Model (no MCV coverage term)	4252.497	4306.261
Model (linear MCV coverage term)	4205.153	4263.804
Model (indicator MCV coverage terms)	4202.318	4280.519
Model (restricted cubic splines for MCV coverage)	4201.080	4279.281

In general, the categorization of MCV coverage that was prospectively defined and used in the base case (1-60%, 60-80%, 80-90%, 90-95%, 95-100%) also performed quite well. However, for the particular data used, several changes to the exact division (shown in italics in **Table S3B**) performed better in terms of minimizing the AIC and BIC. For example, changing the 80% cutoff to 70% or 75% and/or changing the 90% cutoff to 85% all improved both the AIC and BIC.

Table S3B. Alternate categorization of MCV coverage

Specification	AIC	BIC
Model (no MCV coverage term)	4252.497	4306.261
Model (indicator MCV coverage, 1-60, 60-80, 80-90, 90-95, 95-100)	4202.318	4280.519
Model (indicator MCV coverage, 1-20, 20-40, 40-60, 60-80, 80-100)	4204.893	4283.094
Model (indicator MCV coverage, 1-10, 10-80, 80-90, 90-95, 95-100)	4225.608	4303.809
Model (indicator MCV coverage, 1-20, 20-80, 80-90, 90-95, 95-100)	4218.788	4296.988
Model (indicator MCV coverage, 1-30, 30-80, 80-90, 90-95, 95-100)	4217.276	4295.477
Model (indicator MCV coverage, 1-40, 40-80, 80-90, 90-95, 95-100)	4216.87	4295.07
Model (indicator MCV coverage, 1-50, 50-80, 80-90, 90-95, 95-100)	4212.321	4290.522
Model (indicator MCV coverage, 1-60, 60-70, 70-90, 90-95, 95-100)	4207.591	4285.792
Model (indicator MCV coverage, 1-60, 60-70, 70-80, 80-90, 90-100)	4203.739	4281.939
Model (indicator MCV coverage, 1-60, 60-70, 70-85, 85-95, 95-100)	*4201.444*	*4279.645*
Model (indicator MCV coverage, 1-60, 60-80, 80-85, 85-95, 95-100)	*4200.135*	*4278.336*
Model (indicator MCV coverage, 1-60, 60-75, 75-85, 85-95, 95-100)	*4197.825*	*4276.026*

However, even with the categorization of MCV coverage that produced the biggest improvement in AIC/BIC compared to the pre-specified categorization, the direction and significance of all regression coefficients remain largely unchanged compared to the base case. **Table S3C** shows results of this comparison for the linear regression of logged under-5 measles mortality as a function of categorical coverage indicators and control variables.

Table S3C. Comparison of regression coefficients under alternate categorization of MCV coverage levels*

Best Specification (AIC/BIC)			Base Case Specification		
**Independent variables**	**Coefficient**	**P-value**	**Independent variables**	**Coefficient**	**P-value**
MCV coverage of 1-59%	-0.240	0.514	MCV coverage of 1-59%	-0.236	0.523
MCV coverage of 60-74%	**-1.458**	**0.001**	MCV coverage of 60-79%	**-1.639**	**0.000**
MCV coverage of 75-84%	**-2.042**	**0.000**	MCV coverage of 80-89%	**-2.298**	**0.000**
MCV coverage of 85-94%	**-2.623**	**0.000**	MCV coverage of 90-94%	**-2.576**	**0.000**
MCV coverage of >=95%	**-2.977**	**0.000**	MCV coverage of >=95%	**-2.924**	**0.000**
ICD-8 coding system	0.467	0.169	ICD-8 coding system	0.499	0.141
ICD-9 coding system	0.688	0.185	ICD-9 coding system	0.731	0.159
ICD-10 coding system	1.241	0.062	ICD-10 coding system	**1.321**	**0.048**
Year	**-0.119**	**0.000**	Year	**-0.117**	**0.000**
Two doses of MCV	-0.374	0.168	Two doses of MCV	-0.396	0.146
Crude birth rate	**3.364**	**0.000**	Crude birth rate	**3.450**	**0.000**
Urban	**0.077**	**0.011**	Urban	**0.078**	**0.010**
Population density	-1.199	0.144	Population density	-1.302	0.114
Per-capita GDP	-0.380	0.497	Per-capita GDP	-0.464	0.411
Background mortality rate	0.289	0.081	Background mortality rate	0.300	0.070
Constant	225.874	0.000	Constant	223.175	0.000

*Results shown for linear regression of logged under-5 mortality

For ease of interpretation, we computed the % reduction compared to country-years with no MCV coverage implied by the coefficients at each MCV coverage levels as 100 * [1-exp(β)], where β is the regression coefficient for a particular coverage indicator. For example, with the best specification, MCV coverage of 85-94% produces a 93% reduction (i.e., 100 * [1-exp(-2.623)]) compared to a 92% reduction for a coverage level of 90-94% with the base case specification.

While the dependent variable is measles mortality in children under 5, the base case uses MCV coverage in 12-24 month-olds lagged by 1 year. It is therefore possible that vaccination coverage in prior years (i.e., vaccinated 2 year-olds that are now 4 year-olds) have an effect on under-5 mortality as well. At the same time, if case fatality from measles is higher in younger children, including coverage levels for somewhat older children may attenuate the estimated relationship. We use lagged 5-year average MCV coverage in an alternate model specification. In fact, we considered lags in two ways. First, we constructed averages based on all observations within a 5-year range. For example, if data were only available for periods 2 years and 4 years prior, then only these two observations were used to construct the lagged average. Second, we constructed averages requiring that all MCV coverage values be present in the previous 5-year range. The first approach preserves sample size but makes the exact definition of the lagged average harder to interpret. The second approach maintains a clear definition of the lagged average, but loses sample size, and selects certain countries with longer and more continuous data series (see **Table S1D** above). Compared to the base-case specification, the main findings of the regression were similar in the alternative specifications, although the magnitude of the impact of MCV coverage above 80% attenuated under the alternatives (**Table S3D**).

Table S3D. Comparison of regression coefficients: 5-year average MCV coverage vs. 1-year lags*

5-Year Average MCV Coverage Specification			Base case Specification		
**Independent variables**	**Coefficient**	**P-value**	**Independent variables**	**Coefficient**	**P-value**
MCV coverage of 1-59%	-0.654	0.078	MCV coverage of 1-59%	-0.236	0.523
MCV coverage of 60-79%	**-1.499**	**0.000**	MCV coverage of 60-79%	**-1.639**	**0.000**
MCV coverage of 80-89%	**-2.165**	**0.000**	MCV coverage of 80-89%	**-2.298**	**0.000**
MCV coverage of 90-94%	**-2.050**	**0.000**	MCV coverage of 90-94%	**-2.576**	**0.000**
MCV coverage of >=95%	**-2.271**	**0.000**	MCV coverage of >=95%	**-2.924**	**0.000**
ICD-8 coding system	0.667	0.053	ICD-8 coding system	0.499	0.141
ICD-9 coding system	0.983	0.067	ICD-9 coding system	0.731	0.159
ICD-10 coding system	**1.648**	**0.017**	ICD-10 coding system	**1.321**	**0.048**
Year	**-0.146**	**0.000**	Year	**-0.117**	**0.000**
Two doses of MCV	-0.270	0.342	Two doses of MCV	-0.396	0.146
Crude birth rate	**3.408**	**0.000**	Crude birth rate	**3.450**	**0.000**
Urban	**0.064**	**0.039**	Urban	**0.078**	**0.010**
Population density	-0.998	0.232	Population density	-1.302	0.114
Per-capita GDP	-0.237	0.679	Per-capita GDP	-0.464	0.411
Background mortality rate	0.232	0.168	Background mortality rate	0.300	0.070
Constant	277.507	0.000	Constant	223.175	0.000

5-Year Average MCV Coverage Specification (any year in 5-year range without MCV coverage causes observation to drop) n=791		
**Independent variables**	**Coefficient**	**P-value**
MCV coverage of 1-59%	**-1.190**	**0.004**
MCV coverage of 60-79%	**-1.711**	**0.000**
MCV coverage of 80-89%	**-2.535**	**0.000**
MCV coverage of 90-94%	**-2.126**	**0.000**
MCV coverage of >=95%	**-2.294**	**0.000**
ICD-8 coding system	0.451	0.212
ICD-9 coding system	-0.046	0.942
ICD-10 coding system	0.781	0.314
Year	-0.067	0.073
Two doses of MCV	-0.417	0.177
Crude birth rate	**2.281**	**0.020**
Urban	**0.083**	**0.013**
Population density	-1.423	0.135
Per-capita GDP	-0.741	0.288
Background mortality rate	**1.537**	**0.003**
Constant	120.798	0.097

*Results shown for linear regression of logged under-5 mortality

Computing percentage reductions in mortality under different coverage levels as above, we found that the two alternative 5-year lagged specifications predicted reductions in measles deaths at MCV coverage of 85-94% of 87% or 88% compared to 92% in the base case specification.

Supporting Information S4. Sensitivity analysis: alternative regressions among country subsets defined by income or region

We estimated our main model specification on subsets of our data, confining our analysis to countries with higher GDPs or countries outside of less developed regions. For all countries in the analysis, we compared their year 2000 GDP per-capita (Penn World Tables 6.2, purchasing power parity, Laspeyres) to two thresholds I$7,000 and I$5,000. In a second set of regressions, we also excluded countries from Latin America or from Eastern Europe and the Former Soviet Union. We found that in wealthier countries in our sample (>I$7,000), the impact of MCV coverage (especially above 80%) attenuated slightly compared to the base case analysis **(Table S4A**). By contrasting the MCV coefficients from the >I$7,000 and >I$5,000 regressions with the base case, we see that the impact of vaccination was strongest in lower income countries, especially in the range between I$5,000 and I$7,000.

Table S4A. Regression coefficients excluding countries with low per-capita GDP*

	Per-capita GDP >I$7,000		Per-capita GDP >I$5,000		Base case	
**Independent variables**	**Coefficient**	**P-value**	**Coefficient**	**P-value**	**Coefficient**	**P-value**
MCV coverage of 1-59%	-0.317	0.404	-0.274	0.466	-0.236	0.523
MCV coverage of 60-79%	**-1.642**	**0.000**	**-1.713**	**0.000**	**-1.639**	**0.000**
MCV coverage of 80-89%	**-2.057**	**0.000**	**-2.372**	**0.000**	**-2.298**	**0.000**
MCV coverage of 90-94%	**-2.449**	**0.000**	**-2.616**	**0.000**	**-2.576**	**0.000**
MCV coverage of >=95%	**-2.683**	**0.000**	**-2.948**	**0.000**	**-2.924**	**0.000**
ICD-8 coding system	0.502	0.152	0.452	0.186	0.499	0.141
ICD-9 coding system	0.629	0.244	0.675	0.200	0.731	0.159
ICD-10 coding system	**1.705**	**0.016**	1.306	0.056	**1.321**	**0.048**
Year	**-0.120**	**0.000**	**-0.118**	**0.000**	**-0.117**	**0.000**
Two doses of MCV	**-0.663**	**0.021**	-0.419	0.132	-0.396	0.146
Crude birth rate	**4.356**	**0.000**	**3.372**	**0.000**	**3.450**	**0.000**
Urban	**0.066**	**0.043**	**0.094**	**0.003**	**0.078**	**0.010**
Population density	-1.004	0.295	**-1.935**	**0.031**	-1.302	0.114
Per-capita GDP	-0.160	0.827	-0.452	0.444	-0.464	0.411
Background mortality rate	0.136	0.440	0.297	0.074	0.300	0.070
Constant	223.247	0.000	225.573	0.000	223.175	0.000

*Results shown for linear regression of logged under-5 mortality

For ease of interpretation, we computed the % reduction compared to country-years with no MCV coverage implied by the coefficients at each MCV coverage levels as 100 * [1-exp(β)], where β is the regression coefficient for a particular coverage indicator. For example, the model estimated on countries with per-capita GDPs of >$7,000 and >$5,000 predict reductions in measles deaths of at MCV coverage of 85-94% of 91% and 93% respectively, compared to 92% with the base case specification.

It also appears that in Latin American and Eastern European and Former Soviet countries the impact of MCV was strongest (**Table S4B**), although differences were substantively negligible. Computing percentage reductions in mortality under different coverage levels as above, we found that the model estimated on countries excluding those in Latin America or excluding Eastern European Former Soviet countries each predicted reductions in measles deaths of 92% at MCV coverage of 90-94%, equivalent to the predicted reduction in the base case specification.

Table S4B. Regression coefficients excluding countries from less developed regions

Independent variables	Non-Latin America Coefficient	Non-Latin America P-value	Non-Eastern Europe Former Soviet Coefficient	Non-Eastern Europe Former Soviet P-value	Base case Coefficient	Base case P-value
MCV coverage of 1-59%	-0.793	0.046	-0.126	0.737	-0.236	0.523
MCV coverage of 60-79%	***-2.098***	***0.000***	***-1.502***	***0.000***	***-1.639***	***0.000***
MCV coverage of 80-89%	***-2.458***	***0.000***	***-2.153***	***0.000***	***-2.298***	***0.000***
MCV coverage of 90-94%	***-2.503***	***0.000***	***-2.582***	***0.000***	***-2.576***	***0.000***
MCV coverage of >=95%	***-2.851***	***0.000***	***-2.867***	***0.000***	***-2.924***	***0.000***
ICD-8 coding system	0.442	0.194	0.613	0.077	0.499	0.141
ICD-9 coding system	0.504	0.382	0.687	0.194	0.731	0.159
ICD-10 coding system	***1.387***	***0.047***	***1.453***	***0.037***	***1.321***	***0.048***
Year	***-0.117***	***0.002***	***-0.089***	***0.004***	***-0.117***	***0.000***
Two doses of MCV	0.077	0.793	***-0.662***	***0.020***	-0.396	0.146
Crude birth rate	***2.824***	***0.002***	***4.462***	***0.000***	***3.450***	***0.000***
Urban	***0.085***	***0.007***	0.051	0.100	***0.078***	***0.010***
Population density	-1.915	0.315	-0.893	0.296	-1.302	0.114
Per-capita GDP	-0.279	0.669	-1.017	0.140	-0.464	0.411
Background mortality rate	0.592	0.296	0.249	0.132	0.300	0.070
Constant	223.647	0.003	168.935	0.003	223.175	0.000

In general, excluding poorer countries or those from less developed regions had relatively modest effects on our results. This analysis suggests that our base-case analysis may actually underestimate slightly the potential benefit of increasing MCV coverage in other parts of the world.

Supporting Information S5. Sensitivity analysis: year linear trends versus year fixed effects

The main specification of the model includes calendar year as a linear trend. We find a significant linear trend in year with a slope of -0.117 (Reduction of 11.7% in death rate each year after 1960). As an alternative, we instead used year fixed effects (one dummy variable for each year). In the graph below, the coefficients for each year's fixed effect (and confidence intervals) are plotted. The coefficients can be thought of as the logarithm of the odds ratio and also connote a percent reduction from baseline since the model specification is log-linear. The slope of a line fit through the coefficients (-0.1188) is highly concordant with the year linear trend that was defined prospectively and used in this main base case analysis – suggesting that long-term time patterns are generally captured with the linear trend. Other regression coefficients do not change substantially with the use of the year fixed effect specification (not shown).

Figure S5A. Comparison of year trend versus year fixed effects

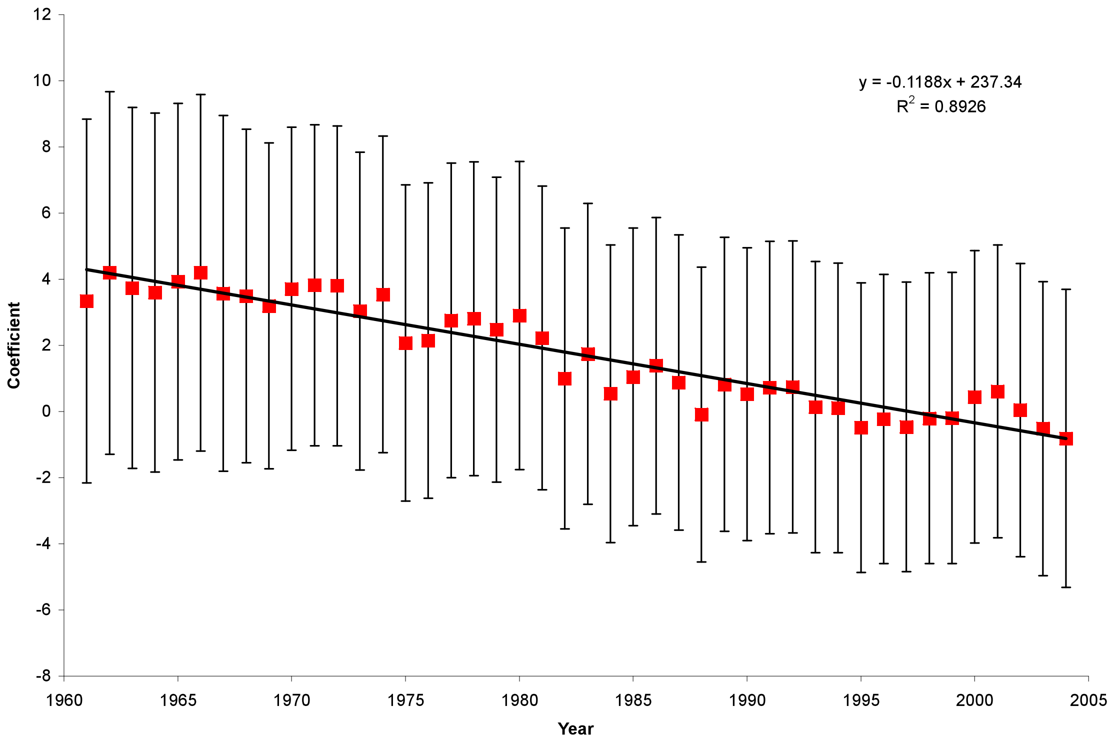

Supporting Information S6. Country-years with no observed measles deaths

For analyses of reductions in measles death rates, the rates were log-transformed (natural log). To deal with years with zero observed deaths (for which the log transformation would be undefined), we replaced the 0 with 0.1 times the minimum observed measles-specific death rate in children under 5. As this was a prospectively-defined but arbitrary choice, we explored the effect of alternative replacement values on model results. We replaced country-years having 0 observed deaths with either the minimum observed measles death rate for children under 5 (i.e., a rate 10 times greater than in the main analysis) or else 0.01 times the minimum observed rate (i.e., a rate 10 times smaller than in the main analysis). We then estimated the model using these alternative outcome variables and compared the resulting coefficients for MCV coverage to the coefficients and 95% confidence intervals from the main analysis. **Figure S6A** shows the results of this sensitivity analysis. The alternative replacements do change the estimated effect, though the magnitude of the change falls within the 95% confidence intervals of the main analysis.

Another alternative would be to use a statistical model for count data such as a Poisson panel regression or a negative binomial panel regression with either fixed or random effects. As Poisson panel regressions are a special case of negative binomial panel regressions in which the mean and variance are assumed to be equal, we estimated negative binomial panel regressions with both fixed and random effects (**Table S6B**). Whereas in the main analysis, MCV coverage of 80% or greater had substantially greater impact than lower levels of coverage, in these alternative models, the effect was more continuous across the coverage levels. Furthermore, at very high coverage levels the reduction compared to country-years with no MCV coverage was estimated to be approximately 80%. The magnitude of the estimated benefit was most consistent with replacing years with no observed deaths with the minimum observed death rates (10 times larger than the main analysis) (**Figure S6A**).

Figure S6A. Comparison of regression models

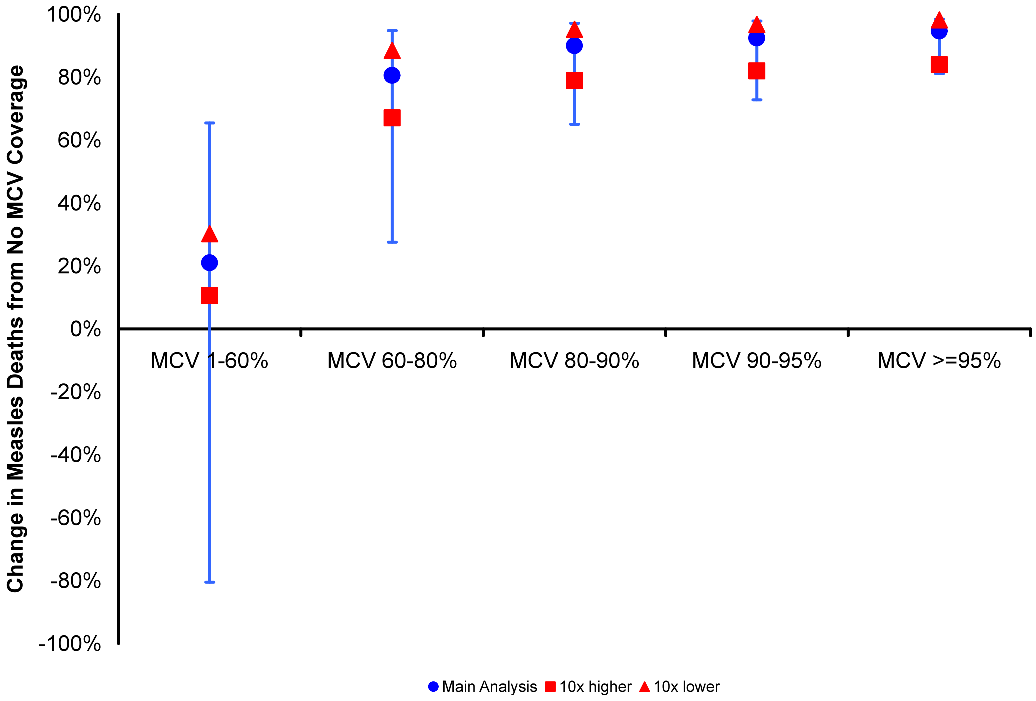

Table S6B. Alternative models: negative binomial panel regressions*

	Conditional country fixed effects			Country random effects		
	Incidence-rate Ratio	95% CI		Incidence-rate Ratio	95% CI	
MCV coverage of 1-59%	0.42	0.35	0.52	0.41	0.34	0.50
MCV coverage of 60-79%	0.28	0.21	0.38	0.27	0.20	0.36
MCV coverage of 80-89%	0.22	0.15	0.33	0.22	0.15	0.32
MCV coverage of 90-94%	0.25	0.17	0.37	0.24	0.16	0.36
MCV coverage of >=95%	0.20	0.13	0.30	0.19	0.12	0.29
ICD-8 coding system	1.26	1.05	1.50	1.28	1.07	1.53
ICD-9 coding system	1.27	0.86	1.85	1.30	0.89	1.92
ICD-10 coding system	1.15	0.61	2.17	1.15	0.61	2.17
Year	0.93	0.91	0.94	0.93	0.92	0.95
Two doses of MCV	0.83	0.60	1.14	0.80	0.59	1.10
Crude birth rate	1.28	0.94	1.73	1.29	0.97	1.74
Urban	0.98	0.97	0.99	0.98	0.97	0.99
Population density	1.79	1.55	2.07	1.66	1.45	1.90
Per-capita GDP	0.79	0.63	0.98	0.73	0.59	0.91
Background mortality rate	0.98	0.90	1.07	0.98	0.91	1.06

* 958 country-years for 42 countries (fixed effects model); 980 country-years for 44 countries (random effects model). Incidence compared to country-years with no measles coverage.
